# Supplementary material for: Deletion of wfs1 Impairs Oligodendrocyte Precursor Cells Dorsal Distribution and Myelination Through the wfs1-hmgcs1 Axis in Zebrafish
Source: Biology (Basel). 2026 Aug 16;15(16):1402. doi: 10.3390/biology15161402 (PMC13510151; doi:10.3390/biology15161402)
Supplement: Supplementary file 1 [file biology-15-01402-s001.zip › File S1-Transcriptome sequencing data and differentially expressed genes (DEGs) in wfs1-- zebrafish at 5 dpf/2.KEGG/wfs1KOvsWT/DOWN/src/dre04146.html]

dre04146


- dre:406803

- Down regulated genes

406803(-2.34145157132947)

- dre:450048

- Down regulated genes

450048(-9.37553654626313)

- dre:110440136

- Down regulated genes

562105(-3.80073354274648) 110440136(-5.33161396140543)

- dre:100003383

- Down regulated genes

100003383\_1(-2.23974139439836)

- dre:406803

- Down regulated genes

406803(-2.34145157132947)

- dre:100093708

- Down regulated genes

100093708(-1.95577016533434)

Close
